# Analysis of Molecular Cytogenetic Alteration in Rhabdomyosarcoma by Array Comparative Genomic Hybridization

**DOI:** 10.1371/journal.pone.0094924

**Published:** 2014-04-17

**Authors:** Chunxia Liu, Dongliang Li, Jinfang Jiang, Jianming Hu, Wei Zhang, Yunzhao Chen, Xiaobin Cui, Yan Qi, Hong Zou, WenJie Zhang, Feng Li

**Affiliations:** 1 Department of Pathology, Shihezi University School of Medicine, Shihezi, Xinjiang, P. R. China; 2 Department of Oncology, Zhongnan Hospital of Wuhan University, Wuhan, Hubei, P. R. China; 3 LU'AN People's Hospital. LU'AN Affiliated Hospital of ANHUI Medical University, LU'AN, Anhui, P. R. China; 4 Department of Pathology, the First Affiliated Hospital, Xinjiang Medical University, Urumqi, Xinjiang, P. R. China; ENEA Italy

## Abstract

Rhabdomyosarcoma (RMS) is the most common pediatric soft tissue sarcoma with poor prognosis. The genetic etiology of RMS remains largely unclear underlying its development and progression. To reveal novel genes more precisely and new therapeutic targets associated with RMS, we used high-resolution array comparative genomic hybridization (aCGH) to explore tumor-associated copy number variations (CNVs) and genes in RMS. We confirmed several important genes by quantitative real-time polymerase chain reaction (QRT-PCR). We then performed bioinformatics-based functional enrichment analysis for genes located in the genomic regions with CNVs. In addition, we identified miRNAs located in the corresponding amplification and deletion regions and performed miRNA functional enrichment analysis. aCGH analyses revealed that all RMS showed specific gains and losses. The amplification regions were 12q13.12, 12q13.3, and 12q13.3–q14.1. The deletion regions were 1p21.1, 2q14.1, 5q13.2, 9p12, and 9q12. The recurrent regions with gains were 12q13.3, 12q13.3–q14.1, 12q14.1, and 17q25.1. The recurrent regions with losses were 9p12–p11.2, 10q11.21–q11.22, 14q32.33, 16p11.2, and 22q11.1. The mean mRNA level of GLI1 in RMS was 6.61-fold higher than that in controls (p = 0.0477) by QRT-PCR. Meanwhile, the mean mRNA level of GEFT in RMS samples was 3.92-fold higher than that in controls (p = 0.0354). Bioinformatic analysis showed that genes were enriched in functions such as immunoglobulin domain, induction of apoptosis, and defensin. Proto-oncogene functions were involved in alveolar RMS. miRNAs that located in the amplified regions in RMS tend to be enriched in oncogenic activity (miR-24 and miR-27a). In conclusion, this study identified a number of CNVs in RMS and functional analyses showed enrichment for genes and miRNAs located in these CNVs regions. These findings may potentially help the identification of novel biomarkers and/or drug targets implicated in diagnosis of and targeted therapy for RMS.

## Introduction

Rhabdomyosarcoma (RMS) is the most common soft tissue sarcoma in children, which has several subtypes including the more aggressive alveolar RMS (ARMS), the more prevalent embryonal RMS (ERMS), and the rare adult variant pleomorphic RMS (PRMS) [1]. Tumorigenesis for some RMSs is recognized, for example, the majority of ARMS tumors (about 85%) are characterized by recurrent translocation between genes encoding for transcription factors FKHR with either PAX3 or PAX7 [2]. The complete genetic etiology underlying RMS development and progression remains unclear.

Array comparative genomic hybridization (aCGH) is a technique that was developed for high-resolution, genome-wide screening of segmental genomic copy number variations [3], [4]. aCGH allows for comprehensive interrogation of hundreds of genomic loci for DNA copy number gains and losses. For the large amount of data generated by high-resolution aCGH, in order to avoid random events of no biologic significance, researchers could deal with the data using various methods, for example GISTIC and waviCGH [5], [6]. DNA copy number changes are common in cancer, and lead to altered expression and function of genes residing within the affected region of the genome. Identification of regions with copy number aberrations, as well as the genes involved, offers a basis for a better understanding of cancer development to provide improved tools for clinical management of cancer, such as new diagnostics and therapeutic targets [7]. Thus, detection of genomic imbalances and identification of these genes can elucidate RMS biology and help identify novel potential biomarkers and targets for clinical therapy.

Traditionally, microarray-based, high-throughput experiments (such as aCGH) produce massive gene lists without consideration of potential relationships among these genes. The gene-by-gene approach often lacks a coherent picture of disease-related pathologic interactions. Bioinformatics has attracted increasing interest in potential gene discovery. For an uploaded gene list, the DAVID bioinformatics resources [8] provide typical gene term enrichment analysis and tools that allow users to condense large gene lists into gene functional groups, visualize many-genes-to-many-terms relationships, categorize redundant and heterogeneous terms into groups, search for interesting and related genes or terms, dynamically view genes from their lists on biopathways, and other functions.

In addition to protein-coding genetic factors, microRNAs (miRNAs) are emerging as key non-protein-coding factors that affect the regulation of gene expression. Increasing evidence suggests that miRNAs participate in nearly all important biological processes, and miRNA dysfunctions are associated with various diseases [9]. Analyses of several human cancers have identified miRNA signatures associated with initiation, progression, diagnosis, or prognosis of tumors [10].

In the present study, high-resolution aCGH was used to identify the potential alterations that were involved in RMS pathogenesis. Genes and miRNAs that located in the altered genomic regions were identified. Finally, tools of DAVID [8] and TAM [11] were used to perform functional enrichment analysis for the identified genes and miRNAs, respectively.

## Materials and Methods

### Ethics Statement

Written informed consent was obtained from all participating patients before enrollment in the study. This study was approved by the institutional ethics committee at the First Affiliated Hospital of Shihezi University School of Medicine and conducted in accordance with the ethical guidelines of the Declaration of Helsinki.

### Samples

Thirty nine formalin-fixed paraffin-embedded (FFPE) RMS samples were selected from archives of the Department of Pathology of the First Affiliated Hospital, Shihezi University School of Medicine and The First Affiliated Hospital of Xinjiang Medical University, China. All patients were Chinese. Each paraffin block was checked to confirm the presence of tumor cells (at least 90%) prior to sectioning and DNA/RNA extraction. This sample set included 15 cases of ARMS, 22 cases of ERMS, and 2 cases of PRMS. The clinical characteristics of patients enrolled in this study and fusion gene status are shown in Table 1. The first 20 samples had been detected by aCGH, including 10 ERMS, 9 ARMS, and 1 PRMS. Fourteen normal muscle tissues were available as controls in QRT-PCR. Two RMS cell lines RD (ERMS) and PLA-802 (ARMS) were obtained from the Biological Technology Co., Ltd. (Fu Xiang, Shanghai, China).

**Table 1:** Clinical characteristics and fusion gene statue of 39 patients with RMS cases.

Case	Sex	Age(year)	Location	subtype	Grade	Stage	Status	Fusion gene	aCGH	QRT-PCR
1	F	8	Nasopharynx	ERMS	G3	4	Dead	-	Y	T
2	F	18	Right lower leg	ERMS	G3	3	NA	-	Y	N
3	M	16	scrotum	ERMS	G3	4	Dead	-	Y	O
4	M	3	Pelvic	ERMS	G2	2	Dead	-	Y	N
5	F	3	Nasopharynx	ERMS	G2	3	Dead	-	Y	T
6	F	13	Left parotid	ERMS	G3	2	Alive	-	Y	T
7	M	40	Nasal cavity	ERMS	G3	2	Dead	-	Y	T
8	F	42	Retroperitoneum	ERMS	G3	3	Dead	-	Y	T
9	M	4	Epididymis	ERMS	G3	2	Alive	-	Y	T
10	F	5	Lymph node	ERMS	G3	4	Dead	-	Y	T
11	F	3	Upper lip	ARMS	G3	2	NA	PAX3-FKHR	Y	T
12	M	28	Right jaw	ARMS	G3	4	Dead	PAX7-FKHR	Y	T
13	F	46	Left atrium	ARMS	G3	2	Dead	PAX7-FKHR	Y	T
14	F	5	Right thigh	ARMS	G3	3	Dead	PAX3-FKHR	Y	N
15	M	18	Left forearm	ARMS	G2	3	Dead	PAX3-FKHR	Y	T
16	F	14	Left parotid	ARMS	G3	2	Dead	PAX3-FKHR	Y	N
17	F	56	Left orbit	ARMS	G3	4	Dead	PAX3-FKHR	Y	N
18	M	16	Left thigh	ARMS	G3	3	Dead	PAX3-FKHR	Y	T
19	M	16	Lumbar	ARMS	G3	4	Dead	-	Y	T
20	M	48	Buttocks	PRMS	G3	3	Dead	-	Y	N
21	M	44	Left hip	PRMS	G3	3	Alive	-	N	T
22	M	2	Buttocks	ERMS	G3	4	Dead	-	N	T
23	M	2	Right orbital	ERMS	G3	2	Dead	-	N	T
24	M	4	bladder	ERMS	G3	2	Dead	-	N	O
25	M	19	laryngeal	ERMS	G3	2	Dead	-	N	T
26	M	29	prostate	ERMS	G3	3	Dead	-	N	T
27	M	20	left spermatic cord	ERMS	G3	2	Alive	-	N	T
28	M	13	testis	ERMS	G3	2	Dead	-	N	O
29	F	41	Retroperitoneum	ERMS	G3	3	Dead	-	N	T
30	F	50	left groin	ERMS	G3	4	NA	-	N	T
31	M	3/4	bladder	ERMS	G3	4	NA	-	N	O
32	M	29	gingiva	ERMS	G3	4	Dead	-	N	O
33	F	18	forehead	ERMS	G3	3	Dead	-	N	O
34	F	2	right infraorbital	ARMS	G3	4	Dead	-	N	O
35	F	14	left auricle	ARMS	G3	2	Dead	-	N	T
36	F	22	Right cheek	ARMS	G3	4	Dead	-	N	T
37	M	68	Left chest	ARMS	G3	3	Dead	PAX3-FKHR	N	T
38	M	15	Right forearm	ARMS	G3	3	Dead	PAX3-FKHR	N	T
39	F	3	left neck	ARMS	G3	4	Dead	PAX3-FKHR	N	T

Note: F: Female, M: Male, NA: not available, Y: Yes, N: No, T: Two (including GLI1 and GEFT), O: only GEFT.

### aCGH

Isolation of genomic DNA (gDNA) from tumor tissues was completed using QIAamp DNA FFPE tissue kit following manufacturer protocols (Qiagen, Germany). The gDNA from the cell lines was isolated using the DNeasy blood and tissue kit (Qiagen, Germany). aCGH experiments were performed using standard NimbleGen protocols (NimbleGen Arrays User's Guide: CGH Analysis v5.1). We used pooled male and female reference gDNA provided by NimbleGen for comparison of male and female patient DNA samples. Tumor DNA fragments and digested references were labeled with Cy3 and Cy5, respectively.

DNA was combined with 40 µL of diluted Cy3-random nonamers and water to a total volume of 80 µL, denatured at 98°C for 10 min, and immediately cooled on ice. This solution was then combined with 20 µL of dNTP/Klenow Master Mix, which was mixed well by pipetting up and down, and incubated for 2 h at 37°C. After clean-up, reference and tumor DNA probes were mixed and hybridized onto a Roche NimbleGen 3×720 K probe platform for 48 h at 42°C. This platform has genome-wide probe spacing at approximately every 2509 bp. After hybridization, array slides were washed and dried in a NimbleGen Microarray Dryer.

### Data processing and analysis

The arrays were scanned using MS200 scanner (NimbleGen) with 2 µm resolution, and fluorescent intensity data was extracted with NimbleScan 2.6 software. The hybridization controls (STC, Sample Tracking Controls) which were used to confirm that the correct samples were hybridized to each array.

For each spot on the array, the log_2_ratios of the Cy3-labeled test sample versus Cy-5 reference sample were computed. Before normalization and segmentation analysis, spatial correction was applied, which corrected position-dependent non-uniformity of signals across the array. Specifically, locally weighted polynomial regression (LOESS) is used to adjust signal intensities based on X, Y feature position [12]. Normalization was then performed using the q-spline method [13], compensated for inherent differences in signal between the two dyes, followed by segmentation using the CNVs calling algorithm segMNT [14]. The segMNT algorithm identified CNVs using a dynamic programming process that minimizes the squared error relative to the segment means, which showed better performance than the DNA copy algorithm [15]. The segments with |mean log_2_ratio| ≥0.25 and at least 5 consecutive probes were retained, |log_2_ratio|<0.25 represented “unchanged”. Mean log_2_ratios of all probes in a chromosome region ≥0.25 were classified as genomic gains, and mean log_2_ratios ≥1.0 were classified as amplification. Meanwhile, mean log_2_ratios ≤–0.25 were regarded as losses, and mean log_2_ratios ≤–1.0 were regarded as deletions [16].

### Real-time quantitative polymerase chain reaction (QRT-PCR)

QRT-PCR was used to validate the results of aCGH. The total RNA was extracted from RMS tissues and 14 cases normal muscle tissues as controls using RNeasy FFPE Kit (QIAGEN), and the total RNAs from all the samples were treated with DNAse I, and transcribed to single-stranded cDNA by reverse transcription using QuantiTect Reverse Transcription Kit (QIAGEN). A house keeping mRNA, ACTB (Hs_ACTB_2_SGQuantiTect Primer Assay, QT01680476), was assessed in all samples. The normal muscle tissues were used as control. The Primers GLI1 (QT00060501) and GEFT (QT00202916) genes were from QuantiTect Primer Assays (QIAGEN). The reaction was carried out on the ABI 7500 Real-Time PCR thermocycler (Applied Biosystems) using Quantifast SYBR Green PCR Kit (QIAGEN). The following thermal cycling program was applied: 5 min at 95°C, 40 cycles of 10 s at 95°C and 30 s at 60°C. Data were normalized for ACTB expression using comparative threshold cycle method. All PCRs were done in triplicates. Cycle threshold (Ct), the fractional cycle number at which the amount of amplified target reached a fixed threshold, was determined. ΔCt values were calculated by subtracting the ACTB Ct values from the target gene Ct values (ΔCt = Ct (GLI1 or GEFT gene in RMS/normal muscle sample) - Ct (ACTB gene in RMS/normal muscle sample)). Expression level was determined as 2-ΔCt.

### Genomic map of the aberrant regions in chromosomes

A genomic map of the aberrant regions was created using Circos [17], which is a software package for displaying genomic data. Circos is a command-line application written in Perl, which can be easily deployed on any system for which Perl is available (http://www.cpan.org/ports/). Inputs were GFF-style data files and Apache-like configuration files, both of which can be easily generated by automated tools.

### DAVID analysis

DAVID (which can be freely accessed at http://david.abcc.ncifcrf.gov/) is a web-based online bioinformatics resource that provides tools for the functional interpretation of large lists of genes/proteins. Amplification or deletion genes were subjected to separate cluster analyses. Each gene set was entered into the DAVID functional annotation clustering tool, which generated clusters of genes based on the similarity of the functional terms assigned to each gene. The clusters were then ranked according to scores of each term, with the higher ranked clusters selected for analysis. Within each cluster, the lowest P value (P value <0.05) was selected as a representative functional term.

### TAM analysis

The genomic coordinate data of miRNAs were downloaded from the miRBase (Release 19). The miRNAs were mapped to their corresponding amplification and deletion regions by an in-house Java program. TAM (http://cmbi.bjmu.edu.cn/tam) was used to identify the enriched functions for the miRNAs within the above regions. Within each cluster, the lowest P value (P value <0.05) was selected as a representative functional term.

### Statistical analysis

SPSS software package (Version 17, Chicago, IL) was used for statistical analyses. Independent sample t test was used to evaluate differences in mRNA expression of GLI1 or GEFT between RMS groups and normal muscle tissues. Differences with a p value of <0.05 were considered statistically significant.

## Results

### Genomic profiles of RMS

**Figure 1:**
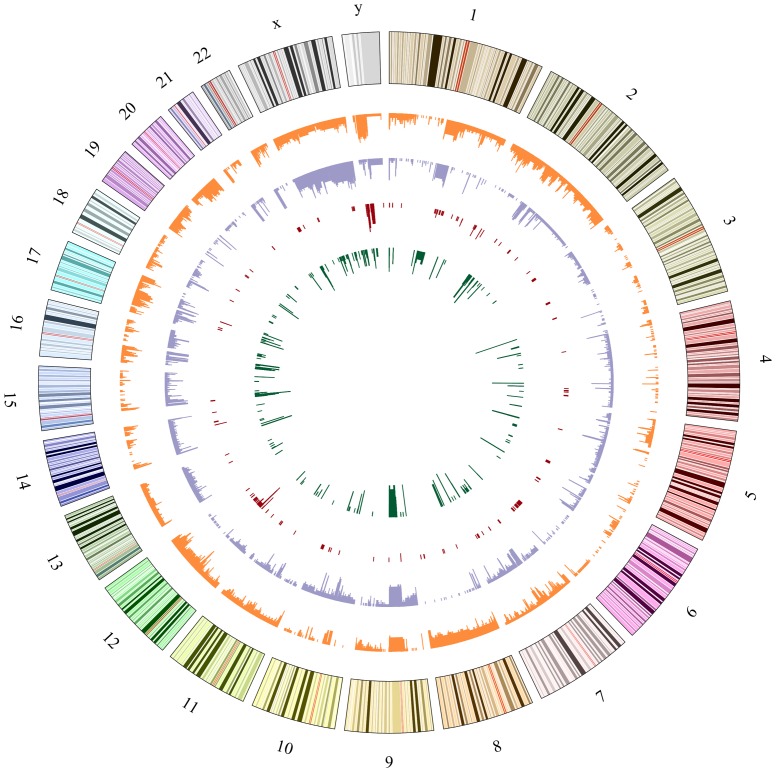
Genomic map of the aberrant regions in 20 cases human RMS chromosomes. The first (outer) circle represents the human chromosome. From the second to the inner, circles highlight the gain regions in orange, the loss regions in purple, the amplification regions in red, and the deletion regions in green.

**Figure 2:**
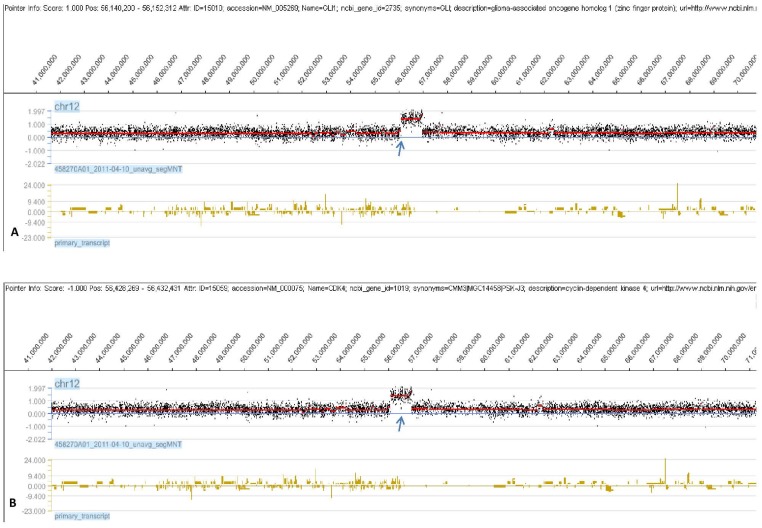
12q13.3–q14.1 region are amplified in RMS. X-axis: 12 chromosome; Y-axis: the sample chip hybridization signal Log2 ratio value. Fig. 2A. GLI1 is amplified in RMS. Fig. 2B. CDK4 is amplified in RMS.

aCGH analysis was carried out to identify genomic alterations in 20 RMS cases, and every RMS tumor displayed copy number changes with gains and losses. Figure 1 shows genomic map of the aberrant regions in human RMS chromosomes. The recurrent region was defined as a region that had a frequency of over 50% of cases. Based on this definition, recurrent regions of gain were 12q13.3, 12q13.3–q14.1, 12q14.1, and 17q25.1. Recurrent regions of loss were 2q14.1, 9p12–p11.2, 9q12, 14q32.33, 16p11.2, and 22q11.1 (Table 2). Then, we listed the frequency of over 20% of cases in regional amplification or deletion on chromosomes (Table 3). The regions of amplification were 12q13.12, 12q13.3, and 12q13.3–q14.1, and 12q14.1. The regions of deletion were 1p21.1, 2q14.1, 5q13.2, 7q35, 8p23.1, and 9q12. Chromosome region 12q13.3–q14.1 was a recurrent region in gain and amplification at 60% and 30%, respectively. The GLI1, GEFT, OS9, CDK4, 9-Mar, MARS, DTX3, and CYP27B1 genes were located on chromosome 12q13.3–q14.1 (Figure 2).

**Table 2:** The most frequent chromosome regions of gain and loss in 20 RMS cases and the genes in these regions.

Change	Chromosome region	Start	End	Frequency(%)	Genes
Gain	1q21.1	143575259	143587461	50	PDE4DIP
	7q11.23	74036774	74123851	50	WBSCR16
	12q13.3	55311006	55381000	55	BAZ2A, ATP5B, PTGES3
	12q13.3	55945619	55983227	50	R3HDM2
	12q13.3–q14.1	56081248	56403704	60	INHBC, INHBE, GLI1, ARHGAP9, MARS, DDIT3, MBD6, DCTN2, KIF5A, PIP5K2C, DTX3, GEFT, SLC26A10, B4GALNT1, OS9
	12q14.1	56403704	56424571	65	CENTG1
	12q14.1	56424571	56529132	60	TSPAN31, CDK4, 9-Mar, CYP27B1, METTL1, TSFM, AVIL, CTDSP2
	17q12	31545582	31548418	50	CCL3L3, CCL3L1
	17q25.1	70567820	70640329	55	KCTD2, SLC16A5, ARMC7, NT5C
Loss	1 p36.33	32108	614850	55	OR4F5, OR4F3, OR4F29
	1q21.1–q21.2	145934357	148061198	50	NBPF9, NBPF8, NBPF1, PPIAL4, NBPF14, NBPF10, BPF8, NBPF15, FCGR1A
	2q11.1	94705333	94911490	50	ANKRD20B, TEKT4
	2q14.1	113899436	114027154	50	CBWD1, CBWD2, DC36, FOXD4L1
	5q13.2	68941167	70418190	50	SMA4
	5q13.2	68941167	70418190	60	SERF1A, SERF1B, SMN1, SMN2, SMA3, SMA5, BIRC1
	9p12–p11.2	40215255	44195143	50	ZNF658, ZNF658B, KGFLP2, ANKRD20A2, DC36, ANKRD20A3, CNTNAP3B
	9p11.2–p11.1	43680045	44964608	55	KGFLP1
	9q12	67756024	69959212	50	DC36, CBWD3, CBWD1, AQP7P2, ANKRD20A1, FOXD4L4, CBWD5
	10q11.21–q11.22	45984298	49059131	55	PTPN20A, PTPN20B, FRMPD2, SYT15, CTGLF1
	10q11.22	46416831	46591997	50	PPYR1, ANXA8L1, ANXA8
	10q23.2	88731868	88879216	55	GLUD1, FAM35A
	14q32.33	105854795	105891901	60	IGHG1, IGHM, IGHA1, IGHG3, IGHG4
	15q11.2	18896456	19887078	50	GOLGA8C, POTE15, OR4M2, OR4N4
	16p13.11	14815552	16435895	55	ABCC6, NOMO2, NOMO1, NPIP, KIAA0251
	16p13.11	15030934	16435895	50	NTAN1, NOMO3
	16p12.2–p12.1	21652458	21745071	55	OTOA, RRN3
	16p11.2	29273447	33680522	55	BOLA2, BOLA2B, GIYD2, SULT1A4, SULT1A3, TP53TG3, SLC6A8
	17p12	15380244	15438749	50	FAM18B2, CDRT1
	17q12	33377006	33427474	50	TBC1D3C, TBC1D3
	17q21.31	41720893	41755952	50	LRRC37A, NBR2
	21p11.1–q11.1–q11.2	10014550	13336318	50	BAGE4, BAGE3, BAGE5, BAGE
	22q11.1	14434579	14867835	55	ACTBL1, OR11H1

**Table 3:** The most frequent chromosome regions of amplification and deletion in 20 RMS cases and the genes in these regions.

Change	Chromosome region	Start	End	Frequency(%)	Genes
Amplification	12q13.12	47781998	47789120	20	LMBR1L
	12q13.3	56081248	56189193	25	INHBC, INHBE, GLI1, ARHGAP9, MARS
	12q13.3–q14.1	56189193	56403704	30	MARS, DDIT3, MBD6, DCTN2, KIF5A, PIP5K2C, DTX3, GEFT, SLC26A10, B4GALNT1, OS9
	12q14.1	56403704	56424571	35	CENTG1
	12q14.1	56424571	56529132	30	TSPAN31, CDK4, 9-Mar, CYP27B1, METTL1, TSFM, AVIL, CTDSP2
	12q14.1	56595122	56725516	20	XRCC6BP1
Deletion	1p36.13	16759656	17082585	20	NBPF1, NBPF10, MST1,ESPNP
	1p21.1	104000830	104102814	20	AMY2A, AMY1A, AMY1C
	1p13.1	116942100	117006929	30	IGSF3
	2q11.1	94705333	94911490	35	RP11-146D12.2, TEKT4, ANKRD20B
	2q13	112841172,	112923310	40	RGPD5
	2q14.1	113890983	114027154	40	CBWD1, CBWD2, DC36, FOXD4L1
	5q13.2	68897382	70188315	35	SMA3, SMA5, SMA4
	5q13.2	69261508	69442825	30	SMA3, SMA5, SERF1B, SERF1A, SMN2, SMN1
	6p22.1	26945775	27052853	35	GUSBL1, MGC22265
	7q22.1	101904918	102612390	20	POLR2J, HSPC047, RASA4, POLR2J3, POLR2J2
	7q35	143520185	143704617	40	OR2A42, OR2A9P, OR2A7, ARHGEF5, OR2A1, OR2A20P
	8p23.3	7702	116437	35	OR4F21
	8p23.1	7825192	7903832	30	ZNF705B, DEFB109
	8p23.1	11901405	12310679	30	DEFB130, DUB3, FAM90A2P, FAM86B1
	9p24.3	33972	181999	40	FOXD4, DC36, CBWD1
	9p13.1	38954658	40108039	25	CNTNAP3, ZNF658
	9p12	41895789	42234758	40	KGFLP2, KGFLP1
	9p12–p11.2	42305064	44672607	35	ANKRD20A2, DC36, ANKRD20A3, CNTNAP3B, KGFLP1
	9q12	67263948	69959212	40	ANKRD20A1, DC36, CBWD3, CBWD1, AQP7P2, FOXD4L4, CBWD5
	9q12–q13	69959212	70157873	40	CBWD1, CBWD3, DC36, FOXD4L3
	9q13	70157873	70220944	40	PGM5
	10q23.2	88731868	89145885	25	GLUD1, FAM35A, FAM22A
	16p13.11	14815552	14964907	20	ABCC6, NOMO2, NOMO1, NPIP
	16p12.3	18253554	18843122	20	NPIP, NOMO2, NOMO1, SMG1
	16p12.1	21708286	21836038	30	RRN3
	16p11.2	31888699	32000280	30	TP53TG3, SLC6A8
	17q12	31697244	31887302	25	TBC1D3C, TBC1D3, TBC1D3B

### Recurrent copy number alterations in ERMS and ARMS detected by aCGH

We analyzed copy number alterations by examining chromosomal changes in ERMS and ARMS tumors. Table 4 listed chromosomal changes and genes with high-frequencies in ERMS and ARMS. Frequent gains were observed in chromosome regions of 7q11.23, 8q24.3, 12q13.3–q14.1, and 19p13.11 in ERMS; frequent losses were found in 5q13.2, 10q11.21–11.22, and 14q32.33 regions in ERMS. Frequent gains were observed in chromosome regions of 1q21.1 and 12q13.3–q14.1 in ARMS; frequent losses were found in 5q13.2 and 14q32.33 regions in ARMS. Frequent gains were detected in GLI1, GEFT, MARS, DDIT3, DCTN2, KIF5A, PIP5K2C, OS9, and CDK4 (12q13.3–q14.1) in ERMS and ARMS; frequent losses were detected in IGHG1, IGHM, IGHG3, and IGHG4 (14q32.33) in ERMS and ARMS.

**Table 4:** Genes of high-frequency changes in ARMS and ERMS.

Subtype	Change	Frequency (%)	Genes
ARMS	Gain	77.78	CENTG1(12q14.1)
		66.67	GLI1, GEFT, OS9, R3HDM2, INHBC, INHBE, ARHGAP9, MARS, DDIT3, MBD6, DCTN2, KIF5A, PIP5K2C, DTX3, SLC26A10 (12q13.3–q14.1); CDK4, 9/Mar, CYP27B1,TSPAN31, METTL1, TSFM, AVIL, CTDSP2(12q14.1);
		55.56	NOTCH2(1p12); NBPF1, NBPF8, NBPF10, NBPF14, NBPF20, PDE4DIP, EC22L1, NOTCH2NL, PPIAL4(1q21.1); CD8B(2p11.2); SETD8(2p11.1); FBXO25(8p23.3); BAZ2A, ATP5B, PTGES3, LRP1, NXPH4, SHMT2, NDUFA4L2, STAC3(12q13.3); XRCC6BP1(12q14.1); CAND1(12q14.3–q15); OR11H12, POTE14(14q11.1); WHDC1L1(15q11.2); RRN3(16p13.11); EIF3S8(16p11.2); GRAP(17p11.2); KCTD2, SLC16A5, ARMC7, NT5C(17q25.1); ACTBL1(22q11.1)
	Loss	66.67	PDPR(16q22.1)
		55.56	SERF1A, SERF1B, BIRC1, SMN1, SMN2, SMA3, SMA5(5q13.2); FRMPD2(10q11.22); OR4P4, OR4S2, OR4C6(11q11); IGHM, IGHG1, IGHG3, IGHG4(14q32.33); SIGLEC5(19q13.33)
ERMS	Gain	70.00	WBSCR16(7q11.23)
		60.00	LAT2, RFC2, CYLN2, GTF2IRD1, GTF2I(7q11.23); PTK2, DENND3, SLC45A4, GPR20, PTP4A3(8q24.3); HOXC8, HOXC6, HOXC5, HOXC4(12q13.13); BAZ2A, ATP5B, PTGES3, INHBC, INHBE, GLI1, ARHGAP9, MARS, DDIT3,MBD6, DCTN2, KIF5A, PIP5K2C, DTX3, GEFT, B4GALNT1, OS9, CENTG1, TSPAN31, CDK4, CYP27B1, METTL1, TSFM, AVIL, CTDSP2(12q13.3–q14.1); TMEM142A, MORN3, GPR109A, GPR109B(12q24.31); CCL3L3, CCL3L1(17q12); MYO9B, NR2F6, MRPL34, DDA1, GTPBP3, PLVAP, BST2, TXNL6, PGL, BCNP1, DDX49(19p13.11); HCST, TYROBP, LRFN3, ALKBH6(19q13.12)
		50.00	PDE4DIP(1q21.1), FKBP1B, SF3B14, TP53I3(2p23.3); OXER1, HAAO, ZFP36L2, THADA(2p21); NEB(2q23.3); BMPR2, ALS2CR13, ALS2CR15, ALS2CR14, WDR12(2q33.1–q33.2); JTV1, EIF2AK1, USP42, PSCD3, RAC1, KDELR2(7p22.1); ERV3(7q11.21); POM121, NSUN5B, NSUN5C, FKBP6, TBL2, CLDN3, CLDN4, ELN, ZP3, DTX2, UPK3B, POMZP3, PMS2L2(7q11.23); ADAM5(8p11.23); RDH10, STAU2(8q21.11); TMEM67, RAD54B(8q22.1); ODF1(8q22.3); EIF3S3, EXT1(8q24.11); TRIB1(8q24.13); DDEF1(8q24.21); BAI1, ARC, JRK, PSCA, LY6K, CYP11B1, LY6E, HHCM, LY6H, GLI4, TOP1MT, RHPN1, MAFA, MAPK15, SCRIB, SIAHBP1, NRBP2, EPPK1, PLEC1, PARP10, GRINA, SPATC1, GPAA1, CYC1, HSF1, DGAT1, SCRT1, CYHR1, KIFC2, FOXH1, GPT, MFSD3, RPL8, COMMD5 (8q24.3); SCGB1C1, ODF3, BET1L, RIC8A(11p15.5); DDN, PRKAG1, MLL2, LMBR1L(12q13.12); HOXC10, HOXC9(12q13.13); ALDH2(12q24.12); CCL4L2, CCL4L1, TBC1D3C, TBC1D3B, TBC1D3, CCL3L3, CCL3L1(17q12); ATP5H, KCTD2, SLC16A5, NT5C, ARMC7, HN1(17q25.1); FGF22, PRG2, PRTN3, GRIN3B, CNN2, POLR2E, ATP5D, MUM1, RPS15, APC2, PCSK4, REEP6, TCF3, KLF16, NCLN, NFIC, HLRC1, PIP5K1C, TJP3, RAXL1, MATK, DAPK3, EEF2, PIAS4, MAP2K2, SIRT6, EBI3, LRG1(19p13.3); EIF3S4, DNMT1, EDG5, ICAM1, CDKN2D, CNN1, ELOF1, ACP5, ZNF627(19p13.2); SAMD1, PRKACA, ASF1B, LPHN1, CD97, DDX39, PKN1, NOTCH3, CYP4F8, CYP4F3(19p13.12); KLF2, EPS15L1, JAK3, MAST3, PIK3R2(19p13.11); MAG, ETV2, POLR2I (19q13.12); BCL2L1, TPX2, MYLK2, KIF3B(20q11.21); EIF2S2, ASIP, AHCY(20q11.22); TGIF2, C20orf24, SLA2, NDRG3, RPN2 (20 q11.23); B4GALT5(20q13.13)
	Loss	70.00	IGHM, IGHG1, IGHG3, IGHG4(14q32.33);
		60.00	OR4F5, OR4F29, OR4F3(1p36.33); SMA1, SMA2, SMA3, SMA5, SMN1, SMN2(5q13.2); KGFLP1(9p11.2–11.1); CTGLF1, PTPN20A, PTPN20B, FRMPD2, SYT15, PPYR1 (10q11.21–11.22); GLUD1(10q23.2); OR11H12, POTE14(14q11.1); VSIG7, GOLGA8D(15q11.2); ABCC6, NOMO1, NOMO2, NOMO3, RRN3(16p13.11); SULT1A3, SULT1A4, TP53TG3, SLC6A8(16 p11.2); HYDIN(16q22.2); NBR2, LRRC37A, ARL17P1(17q21.31); GGT2, USP41(22q11.21)
		50.00	NBPF10, NBPF1, MST1, ESPNP (1p36.13); IGSF3(1p13.1); NBPF9, NBPF8, PPIAL4, NBPF14, NBPF15 (1q21.1); FCGR1A(1q21.1–q21.2); HIST2H3C, HIST2H2AA, HIST2H4(1q21.2); SRGAP2(1q32.1); LSP1, SETD8(2p11.1); ANKRD20B, TEKT4(2q11.1); CBWD1, CBWD2, DC36, FOXD4L1(2q14.1); GYPB, GYPA(4 q31.21–q31.22); OCLN, SMA3, SMA5, SMA4, BIRC1, GTF2H2(5q13.2); GUSBL1(6p22.1); DEFB103A, SPAG11, DEFB107A, DEFB106A, DEFB104A, DEFB4 (8p23.1); CNTNAP3, ZNF658(9p13.1); KGFLP2, KGFLP1, DC36, CNTNAP3B(9p12–p11.2); CBWD3, CBWD1, AQP7P2, CBWD5(9q12); ANXA8L1, ANXA8(10q11.22); TRIM64(11q14.3); GOLGA8C, POTE15, OR4N4(15q11.2); FAM7A1, ARHGAP11A(15q13.3); RPS17(15q25.2); OR4F4(15q26.3); ABCC6, NOMO1, NOMO2, NOMO3(16p13.11); WWP2, HYDIN(16q22.1); TBC1D3, TBC1D3C, TBC1D3B(17q12); PLEKHM1, ARL17P1, NSF(17q21.31); USP32(17q23.2)

### Recurrent copy number alterations in RMS cell lines detected by aCGH

**Figure 3:**
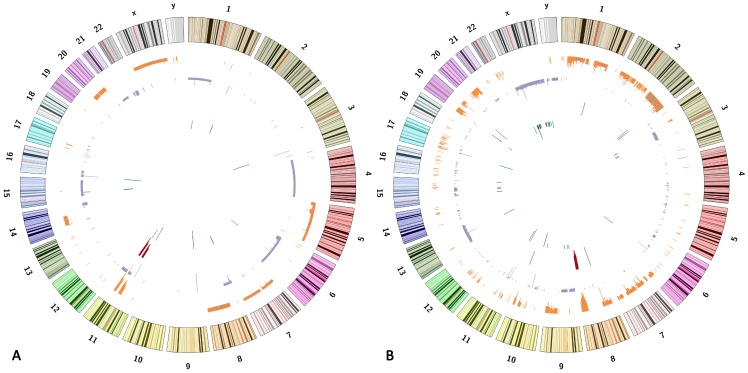
Genomic map of the aberrant regions in a human RMS cell lines chromosomes. The first (outer) circle represents the human chromosome. From the second to the inner, circles highlight the gain regions in orange, the loss regions in purple, the amplification regions in red, and the deletion regions in green. Fig 3A. PLA-802 cell line; Fig 3B. RD cell line.

Chromosome imbalance was detected in the RMS cell line by aCGH. Figure 3A and B show the genomic maps of the aberrant regions in PLA-802 (ARMS) and RD (ERMS), respectively. As shown in Figure 3, frequent chromosomal changes were observed in the two cell lines. It was of interest to note that certain ERMS tissues showed the same chromosomal changes as the ERMS cell line, including gains at 7q11.23, 8q24.3, 19p13.11, 8q24.13, and 8q24.21 regions, and losses at 15q11.2 and 16p11.2 regions, respectively. The 14q32.33 loss and 8p23.1 deletion were identified in both the ARMS cell line and the ARMS tissues.

### GLI1 and GEFT mRNA Expression in RMS tissues and cell lines by QRT-PCR

**Figure 4:**
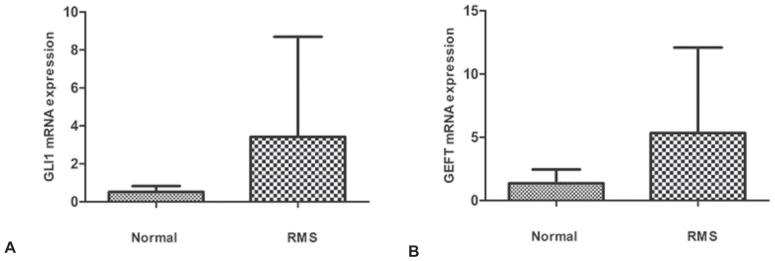
Expression level of GLI1 and GEFT mRNA in RMS samples in comparison with normal muscle tissue. Fig. 4A. Expression level of GLI1 mRNA in RMS compared with normal muscle tissue (3.421+1.034 vs 0.5174+0.083, p = 0.0477). Fig. 4B. Expression level of GEFT mRNA in RMS compared with normal muscle tissue (5.326+1.178 vs 1.359+0.294, p = 0.0354). Columns, the expression level of mRNA in whole RMS samples or normal muscle tissues; bars, SD.

We confirmed the overexpression of GLI1 mRNA in RMS by using QRT-PCR. We compared mRNA expression levels of GLI1 in 26 tumor specimens and GEFT in 33 tumor specimens to normal muscle tissues using real time PCR. To accurately quantify mRNA expression of GLI1 and GEFT, ACTB was similarly amplified as an internal control to normalize the results. As a whole, the mean mRNA level of GLI1 in RMS samples was 6.61-fold higher compared with those in normal muscle tissues, as shown in Figure 4A (3.421+1.034 vs 0.5174+0.083, p = 0.0477). The mean mRNA level of GEFT in RMS samples was 3.92-fold higher compared with those in normal muscle tissues, as shown in Figure 4B (5.326+1.178 vs 1.359+0.294, p = 0.0354).

The mean mRNA level of GLI1 in PLA-802 and RD was lower compared with those in normal muscle tissues, being 0.0006-fold and 0.0076-fold, respectively. The mean mRNA level of GEFT in PLA-802 and RD was lower compared with those in normal muscle tissues, being 0.0015-fold and 0.03-fold, respectively.

### Functional annotation clustering in RMS

**Figure 5:**
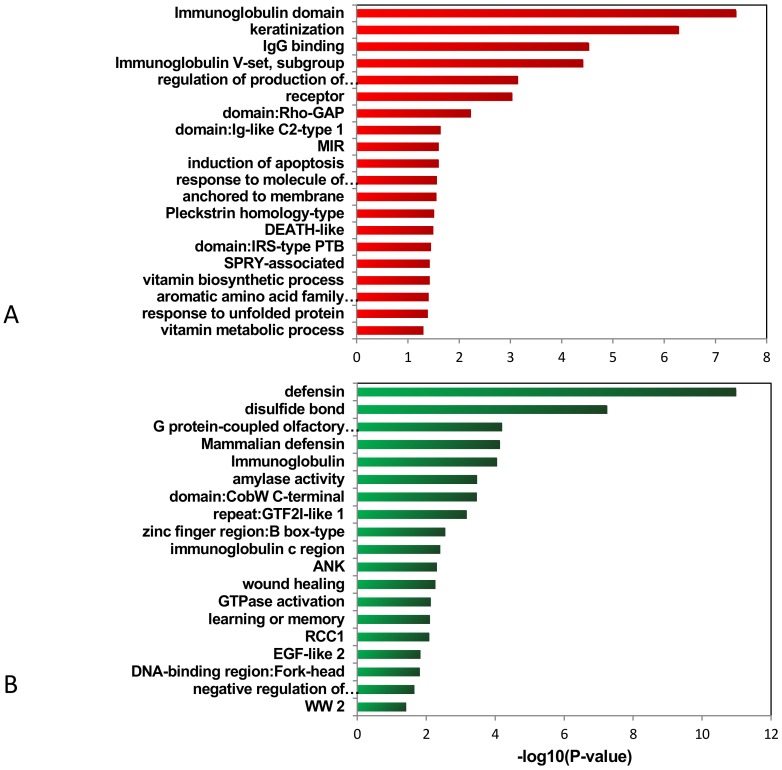
Enriched functions of genes within the amplification regions (A) and deletion regions (B) in RMS.

Given that many genes are biologically related, grouping these highly connected genes by network analysis may reveal underlying functional processes in a manner complementary to standard differential expression analyses. We used DAVID functional annotation clustering to allow biological interpretation in a group level and analysis of the internal relationships among the clustered terms. Figure 5 listed the enrichment values associated with certain categories in RMS. It showed that many gene-enriched functional regions were involved in RMS. The representative amplification genes were related to the immunoglobulin domain, Rho-GAP domain, and induction of apoptosis. The representative deletion genes were related to defensin, amylase activity, wound healing. The functions of regions and genes are listed in Table 5.

**Table 5:** Functional annotation cluster of the genes that located in the chromosome regions of amplification and deletion in 20 RMS cases.

Change	Annotation Cluster	Genes
Amplification	immunoglobulin domain	FGFR2, IGHG1, CD244, IGHG3, IGHG4, VPREB1, LRIG3, LY9, IGHM, PVRL4, UNC5A, IGHA1, FCGR3A, FCGR3B, IFNGR1, NCR2, F11R, BTNL8, MPZ, BTNL9, FLT4, BTNL3, SLAMF7, PALLD, CD84, FCGR2C, LRRN1, TREML4, TREML3, CNTN4
	keratinization	LCE4A, LCE3A, LCE3B, LCE5A, LCE3C, LCE3D, LCE2C, LCE2D, LCE2A, LCE3E
	Rho-GAP	BCR, MYO9B, ARHGAP15, INPP5B, ARHGAP10, ARHGAP9, ARHGAP30
	induction of apoptosis	BID, BCLAF1, BCL2L13,DEDD, TLR2, FASLG, SLAMF7, PLAGL1, MAPK1, MAP3K5, MAPK9, DYRK2, PERP, PHLDA3, PRODH
	Pleckstrin homology-type	GEFT, NM3, NOS1AP, RGPD4, PLEKHH1, SLC26A10, GAB4, DOK3, PSD, FRS3, RANBP1, FRS2, APBA1, ARHGAP9, ARHGAP10, ARHGAP15
	vitamin metabolic process	ALDH8A1, NMNAT3, KYNU, CYP27B1, RBP1, RBP2
	receptor	MET, FGFR2, IGHG1, IGHG3, GPR126, IL27RA, GPR109B, GPR109A, TLR2, IGHM, DGCR2, UNC5A, FCGR3A, FCGR3B, RGR, HTR1F, IFNGR1, LPHN1, F11R, NPBWR2, ESR1, NCR2, CD84, IL20RA, GRM6, OR4N4, OR8B2, OR8B3, TREM1, OR8B4, OR8B8, TREM2, MYO18A, CD244, SCARF2, OR4F3, IRAK3, FCER1G, PTPRE, OPRL1, OR2Y1, ESRRB, OR8G5, FLT4, RTN4R, SNW1, SLAMF7, ITPR1, EPHA3, LMBR1L, NR1I3, LRP1, FCGR2C, TOMM40L, GRK6, OR8D1, OR8D2, IL5RA, FCGR2A, NMBR, LRP3
	response to unfolded protein	DDIT3, ATF6, IFNG, HSPA6, CREB3L4, EDEM1
Deletion	defensin	DEFB130, DEFB108B, DEFB107A, DEFB106A, DEFA5, DEFB103A, DEFB104A, DEFA3, DEFB105A, DEFA1, DEFB109
	immunoglobulin	KIT, IGHG1, IGHG3, PSG2, PSG3, PSG1, IGHM, PSG11, PSG8, PSG7, PSG6, SIGLEC5, FCGR1A, PSG4, IGSF3
	amylase activity	AMY2A, AMY2B, AMY1C, AMY1A
	disulfide bond	MUC4, NOTCH2, KIT, IGHG1, OR2A1, IGHG3, NRG3, IGHG4, OR4F21, OR2A42, OR4C6, OR11H12, IGHM, OR4F29, PSG11, OR4S2, CNTNAP3, CHRNA7, AMY2B, AMY2A, NRXN3, PSG2, OR4M2, OR4F17, PSG3, PSG1, OR4M1, CFC1, PSG8, PSG7, SIGLEC5, PSG6, DEFA5, PSG4, IGSF3, DEFA3, OR4N4, DEFA1, EFNA5, GALNT8, AMY1C, AMY1A, PAM, DRD5, PPYR1, MST1, CNTNAP3B, SFTPA1, OR4F4, OR4F3, FCGR1A, IGHA1, OR4Q3, PLGLB2, PLGLB1, KLK6, KLK7, DEFB130, DEFB107A, DEFB103A, NOTCH2NL, OR11H1, OR4F5, ST8SIA4, SFTPA2, OR4P4
	mammalian defensin	DEFA5, DEFA3, DEFA1, DEFB109
	domain: EGF-like	NOTCH2, NRXN3, CNTNAP3, NOTCH2NL, CNTNAP3B, MUC4
	GTPase activation	TBC1D3C, NF1, ARHGAP27, ARHGAP11A, ARHGAP15, RASA4, TBC1D3, TBC1D3B
	DNA-binding region: Fork-head	FOXD4L4, FOXD4L3, FOXD4L1, FOXD4

### Functional annotation clustering in ARMS and ERMS

**Figure 6:**
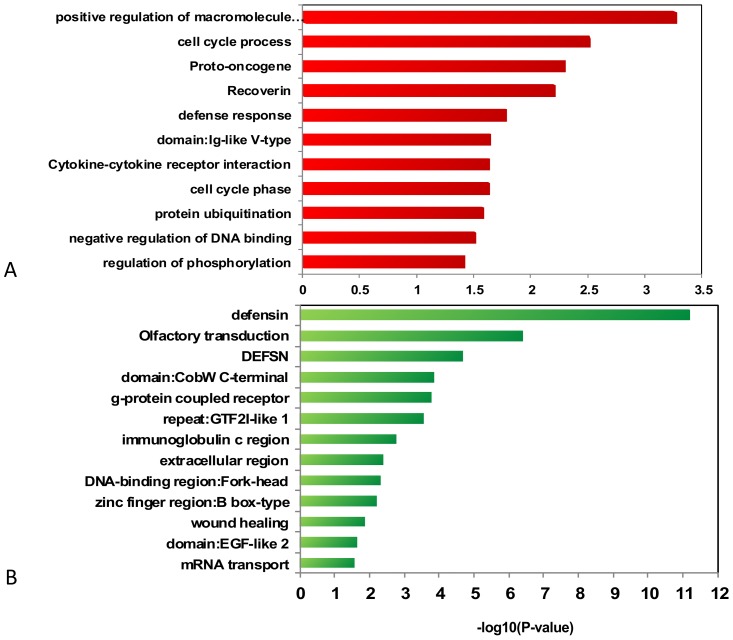
Enriched functions of genes within the amplification regions (A) and deletion regions (B) in ARMS.

**Figure 7:**
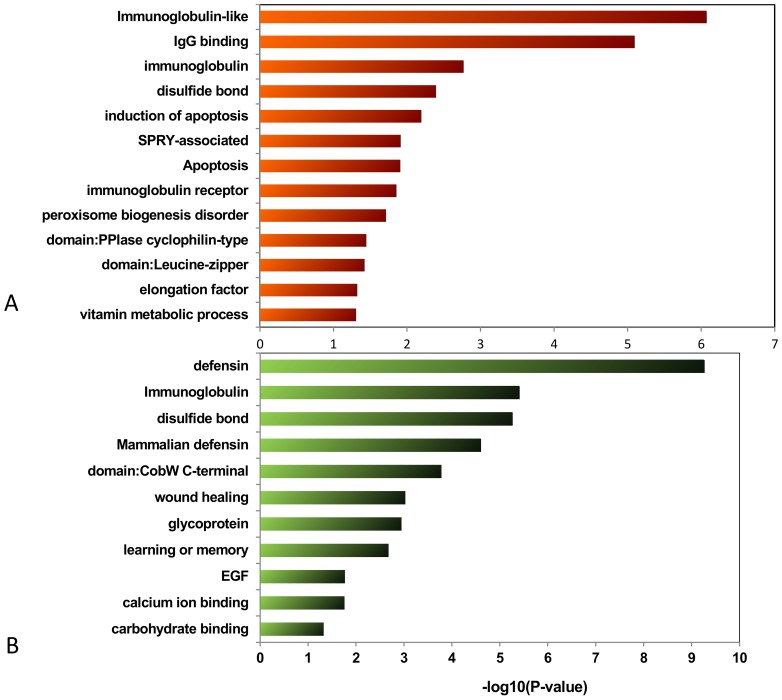
Enriched functions of genes within the amplification regions (A) and deletion regions (B) in ERMS.

We analyzed amplification and deletion regions genes using DAVID in ARMS and ERMS, respectively. Figures 6 and 7 listed the enrichment values associated with certain categories in ARMS and ERMS, respectively. Numerously enriched functions of genes were found within the amplification regions, but they differed between ARMS and ERMS. In ARMS, for example, enriched functions of genes within the amplification regions included cell cycle process and proto-oncogene. Functional annotation clustering amplification of the cell cycle process included CYP27B1, MDM2, CDK4, and high mobility group AT-hook 2 (HMGA2). Functional annotation clustering amplification of proto-oncogene included GLI1, MDM2, CDK4, HMGA2, MET, and DDIT3 (Table 6). In ERMS, enriched functions of genes within the amplification regions included immunoglobulin-like, IgG binding, and induction of apoptosis. Enriched functions of genes were observed within the deletion regions, include defensin, and wound healing, in the two types of RMS. The correlations of these genes and RMS tumorigenesis were previously unknown, and some could have a function in tumorigenesis processes.

**Table 6:** Contrast between ARMS and ERMS in annotation cluster.

Type		Annotation Cluster	Genes
ARMS	Amplification	cell cycle process	CYP27B1, MDM2, CDK4, HMGA2, YEATS4, DDX11, IFNG, UBR2, DDIT3, DCTN2
		proto-oncogene	MET, MDM2, CDK4, HMGA2, GLI1, DDIT3
		positive regulation of macromolecule metabolic process	YEATS4, GRIP1, TFEB, NFYA, CDK4, TRERF1, IL22, DDIT3, GLI1, CCND3, IFNG, MDM2, CAND1, DYRK2
		Cytokine-cytokine receptor interaction	INHBE, MET, IFNG, INHBC, IL26, IL22
	Deletion	defensin	DEFB130, DEFB107A, DEFB106A, DEFB103A, DEFA5, DEFB104A, DEFA3, DEFB105A, DEFA1, DEFB109
		immunoglobulin c region	IGHG1, IGHG3, IGHG4, IGHA1, IGHM
		zinc finger region: B box-type	TRIM64, TRIM49, TRIM74, TRIM73
		mRNA transport	RGPD5, NPIP, POM121, RGPD4, SMG1
		DNA-binding region: Fork-head	FOXD4L4, FOXD4L3, FOXD4L1, FOXD4
ERMS	Amplification	immunoglobulin-like	IGHG1, FGFR2, CD244, IGHG3, IGHG4, VPREB3, VPREB1, LY9, IGHM, PVRL4, UNC5A, IGLV6-57, FCGR3A, FCGR3B, F11R, BTNL8, MPZ, BTNL9, TRGV9, FLT4, BTNL3, SLAMF7, PALLD, SLAMF1, CD84, FCGR2C, IGLV3-25, FCGR2A, IGLC2, IGLC1
		induction of apoptosis	BID, BCLAF1, DEDD, TLR2, FASLG, SLAMF7, BCL2L13, PLAGL1, MAPK1, MAP3K5, MAPK9, PERP, PHLDA3, PRODH
		IgG binding	FCGR2C, FCER1G, FCGR2A, FCGR3A, FCGR3B
		IPR006574: SPRY-associated	BTNL8, BTNL9, TRIM7, TRIM41, BTNL3
		immunoglobulin	IGHG1, IGHG3, IGHG4, FCGR2C, IGLC2, IGHM, IGLC1, IGLV4-3
	Deletion	defensin	DEFB130, DEFB107A, DEFB106A, DEFB103A, DEFA5, DEFB104A, DEFA3, DEFA1, DEFB109
		immunoglobulin	IGHG1, IGHG3, PSG2, PSG3, PSG1, KIT, IGHM, PSG11, PSG8, PSG7, PSG6, SIGLEC5, FCGR1A, PSG4, IGSF3
		mammalian defensin	DEFA5, DEFA3, DEFA1, DEFB109
		IPR006209:EGF	NOTCH2, NRG3, NRXN3, CNTNAP3, NOTCH2NL, CNTNAP3B
		carbohydrate binding	NOMO3, SIGLEC5, SFTPA2, KGFLP2, SFTPA1, GALNT8, NOMO1, KGFLP1, NOMO2
		glycoprotein	AMY2A, AMY1B, AMY1A, AMY1C, GYPB, IGHG1, SLCO6A1, ADCY1, IGHG3, NRG3, GYPE, IGHG4, STRC, GYPA, GGT2, IGHM, PSG11, CNTNAP3, CHRNA7, GPR89A, LRRC37A, MINPP1, NRXN3, PLD5, PSG2, PSG3, PSG1, PSG8, PSG7, SIGLEC5, PSG6, PSG4, IGSF3, UGT2B11, GALNT8, OTOA, PAM, DRD5, PPYR1, MST1, CNTNAP3B, SFTPA1, KIT, OR4F3, FCGR1A, NOMO3, MUC20, NOTCH2NL, NOTCH2, SLC6A8, ST8SIA4, SFTPA2, NOMO1, NOMO2, ABCC6

### miRNA functional enrichment analysis

**Figure 8:**
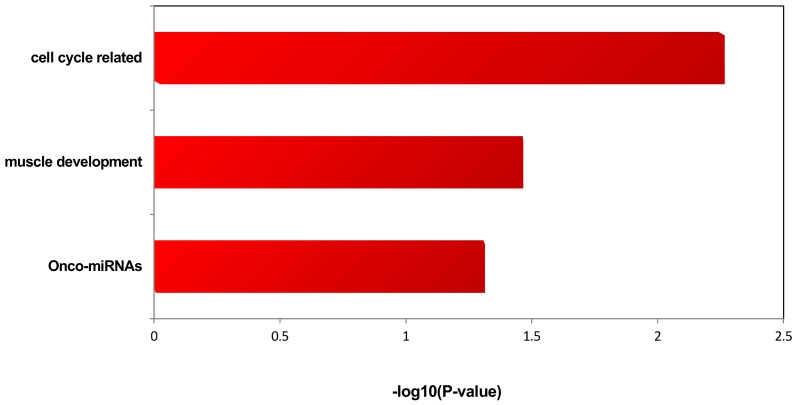
Enriched miRNA functions of miRNAs within the amplification regions in RMS.

Enriched miRNA functions were analyzed for the upregulated and downregulated miRNA in RMS by TAM. The upregulation of onco-miRNA, cell cycle-related miRNA, and muscle development miRNA were associated with RMS, as shown in Figure 8. The regulation of muscle development miRNAs included miR-24, miR-27a, and miR-331. A subset of onco-miRNAs (miR-24, miR-27a, and miR-146b) was associated with RMS (Table 7). No significant results were obtained for the downregulated miRNA in RMS.

**Table 7:** Enriched functions for miRNAs that are located in the chromosomes of amplification in RMS.

Function	miRNA
onco-miRNAs	miR-24, miR-27a, miR-146b
muscle development	miR-24, miR-331, miR-503, miR-27a, miR-185, miR-424
cell cycle-related	miR-24, miR-23a

## Discussion

RMS, the most common soft tissue sarcoma in children, likely results from an imbalance in the proliferation and differentiation of precursor cells during the skeletal myogenesis program. Despite improved understanding of the molecular pathogenesis of RMS in recent decades, patient outcomes remain poor. To increase the accuracy of RMS outcome prediction, efficient molecular markers are needed. An increasing number of evidence shows that gene amplification or deletion is often involved in tumorigenesis and/or tumor progression. Correlations between genomic copy number and gene expression levels have been indicated [18], [19], [20].

In the current study, high-resolution aCGH was used to provide accurate molecular information on the pathogenesis of RMS. Combined with DAVID, we determined the potential relationships of these massive genes, and improved these genes from biological angles and biological interpretation in a network context.

Only a few studies have reported chromosomal changes in frozen RMS or cell lines by aCGH. However, the resolution and number of genes covered by these aCGH chips vary substantially. Using frozen tissues and cell lines as materials, we summarize the results in Table 8. In our study, we used FFPE archival tissues as materials to efficiently detect chromosomal changes in RMS by high-resolution aCGH technique.

**Table 8:** Chromosome imbalance in RMS by aCGH [21]–[26].

Year	Author	Materials	Array	Results
2004	Sandra et al.	2 ARMS cell lines	composing of 58 different genomic DNA fragments	gain: FGR(1p36.2∼p36.1), NRAS(1p13.2), REL(2p13∼p12), MYCN(2p24.1), PGY1(7q21.1), MET(7q31), ABL1(9q34.1), MLL(11q23.1), EMS1(11q13), GARP(11q13.5∼q14), KRAS2(12p12.1), SAS/CDK4(12q13.3), GLI(12q13.2–q13.3), AKT1(14q32.3), RPS6KB1(17q23)loss: PIK3CA(3q26.3), PDGFRA(4q12), EGFR(7p12.3∼p12.1), MET(7q31), FES(15q26.1), BCL2(18q21.3), CCNE1(19q13.1), JUNB(19p13.2), PTPN1(20q13.1∼q13.2)
2006	Myriam et al.	frozen tissue (7 ARMS, 2 ERMS, 1 PRMS)	one containing 57 oncogenes, the other containing 287 genomic clones	gain: FGR, LAMC2, TGFB2, AIB1, TNFRSF6B, TOM, MSH2, GARP, PAK1, PDGFB, CCND2, PTPN1, CSF1R, MYC, PTK2, WNT1, CDK2, ERBB3, SAS/CDK4, AKT2, Chr 22, MYCN, RAF1, ERBB, D17S1670, CBFA2, 8ptelamplification: AKT2, GLI, SAS/CDK4, MYCL1, FGFR1loss: HIC1, D17S125, 22QTEL31, Chr 3, PDGFRA, HRAS
2009	Edoardo et al.	cell lines (4 ERMS, 7 ARMS, 1 PRMS, and 1 RMS of uncertain origin)	cDNA microarray containing 30049 clones	gain: 1p21.3–13.2, 1q12, 6q26–27, 7q21.3–31.1, 9q33.3, 12q15, 19q12, 17q25, 20p13, 20q,1q41(CENPF), 2p24.3(MYCN), 8q24.12(MYC), 20q13.2(BCAS1), 20q13.32(GNAS)amplification: 2p24.3(MYCN), 8p11.23–11.21(FGFR1), 12q13.3(CDK4), 19q12, 20qloss: 3p14.2∼12.2, 4q27∼32.3, 6p25.1∼24.3, 9p24.3∼24.1, 13q14.3
2009	Frederic et al.	57 frozen ARMS tissue	50K and 250K array analysis 2p24 and12q13–q15	amplification: 12q15, 2p24(DDX1,MYCN), 12q13–q14(CDK4, CENTG1, DDIT3, DTX3, MBD6, PIP5K2C 27 human genes)
2010	Daniel et al.	128 frozen RMS(77 ARMS, 51 ERMS)	a BAC array	Gain: 7, 8, 11, 20amplification: 2p24.1 (MYCN), 8p11.2–p11.1 (FGFR1), 12q13.3–14.1 (CDK4), MDM2(12q14.3–q15)
2011	Vera et al.	26 frozen ERMS	2.1million probe platform (2.1M)	gain: 8, 2,11,12,13, 19, 20amplification: 2p21(TTC7A), 2q35(TNS1), 2q14.2(GLI2), 2q36.1(MOGAT1), 5q35.2–q35.3(FGFR4), 11p11.2(CREB3L1, DGKZ), 11q24.2(ROBO3, ROBO4), 12q13.3(IHBC, INHBE, GLI1, ARGHAP9, MARS, DDIT3, MBD6, DCTN2, KIF5A, PIP4K2C, DTX3, GEFT, SLC26A10, B4GALNT1, LRP1, NXPH44, SHMT2, NDUFA412, miR1228)loss: 6,9,10,14,15,16,18,deletion: 1p36.23(RERE), 1q32.1(PPP1R12B), 3p14.2(PTPRG, FHIT), 4q35.1–35.2(F11, ANKRD37, UFSP2, C4orf47, CCDC110, PDLIM3, SORBS2, TLR3, FAM149A, CYP4V2, KLKB1), 9p21.3(CDKN2A/B), 17q11.2(NF1), 22q13.31(ATXN10)

From Table 8, we found that many probes only included a few genes and regions. The focused regions and genes in previous studies include 12q13.3–q14.1, 8p11.2–11.2, and CDK4, MYCN, GLI, MDM2, FGFR1, and FGFR4, respectively [21], [22], [23], [24], [25], [26]. Most of them exists frequent gains and amplifications. Using an aCGH platform to examine a specific subset of 26 frozen ERMS samples, Vera et al. have found that these tumors share a common genomic program with a high frequency of gains at 12q13.3 (about 50%) in ERMS [26]. In this study, we have observed high frequencies of gains at 12q13.3–q14.1 in RMS (about 60%), in ERMS (60%), and in ARMS (66.67%), respectively. The above regions include genes such as GLI1, GEFT, OS9, CDK4, PIP5K2C, and CYP27B1. Edoardo et al. indicated that overexpression of the CDK4 and MYCN genes is involved in RMS tumorigenesis, and CENPF, DTL, MYC, EYA2, and FGFR1 are functionally relevant [23]. Daniel et al. showed that the frequency of many specific amplifications and gains (CDK4 and MYCN) significantly varies between fusion gene-positive ARMS and fusion gene-negative ARMS and ERMS, and CDK4 exhibits a high frequency of amplifications and gains in fusion gene-positive ARMS [25]. However, we found that the frequencies of CDK4 amplifications in ERMS (3/10, 30%) were similar to those in ARMS (3/9, 33.3%). One potential reason for the difference could be resulted from differences in probe resolution, sample quantity, and ethnicity.

GLI1, as well as two other members of the GLI family, is a nuclear mediator of the Hh signaling pathway. Treatment with small-molecule Hh signaling inhibitors inhibits cell proliferation in the ERMS cell lines, which suggested that GLI1 could be an effective therapeutic target for ERMS [27]. Betulinic acid induces apoptosis and inhibits Hh signaling in RMS [28]. GLI1 is expressed at significantly higher levels in ERMS and fusion gene-negative ARMS compared with those in fusion gene-positive ARMS. Targeted inhibition of Hh signaling can be an effective strategy for the development of future RMS treatments and prevention [29]. In this study, we found a high frequency of gain and amplification of GLI1, being 60% and 25%, respectively. By QRT-PCR, we found that the mean mRNA level of GLI1 in RMS samples is higher than that in normal muscle tissues. These findings suggest that GLI1 may play an important role in the pathogenesis of RMS, promising GLI1 as a potential candidate biomarker in treatment of RMS.

GEFT was identified as a novel Rho-family-specific guanine nucleotide exchange factors (GEFs), which is highly expressed in brain, heart, and skeletal muscles [30]. Vera et al. demonstrated that GEFT is amplified in ERMS [26], in keeping with our results as shown in RMS. Although the function of GEFT in tumors is unknown, many members of Rho GEFs exhibit increased abundance or activity in human tumors, and potentially affect cancer progression [31], [32], [33]. In this study, we found that the mean mRNA level of GEFT in RMS samples is higher than that in normal muscle tissues, suggesting that the involvement of GEFT in RMS pathogenesis could be a potential candidate biomarker in the treatment of RMS. To the best of our knowledge, the present investigation is the first study to report on the mRNA expression of GEFT in RMS samples. The specific role of GEFT in RMS needs further research.

MDM2 is an E3 ubiquitin ligase that regulates the protein level of p53 through ubiquitin-dependent degradation [34]. MDM2 is overexpressed in RMS cases [35]. Mitsuru et al. showed that inhibiting the MDM2-p53 pathway with a small-molecule antagonist of MDM2 suppresses tumor growth and induces death of RMS cells, and can be a potential therapy for patients with RMS [36]. Ziad A. et al reported that co-amplification of the CDK4 gene with MDM2 and GLI occurs in human sarcomas, including RMS, Ewing's sarcoma, osteosarcoma, and undifferentiated sarcoma [37]. In the present study, we found that these genes were highly expressed in RMS. Thus, these results are support by the published data.

Furthermore, functional annotation clustering of the expression data readily distinguished genomic alterations in RMS. The high resolution of aCGH combined with human genome database helped in determining the possible target genes present in amplification or deletion regions. By functional annotation clustering, we found many genes involved in tumorigenisis. Many of the abnormalities observed in this study encompass the hallmark chromosomal changes reported in the literature.

By functional annotation clustering, we found that the genes MET, HMGA2, CDK4, MDM2, GLI1, and DDIT3 played a role of proto-oncogene in ARMS. MET oncogene is a unique receptor tyrosine kinase (RTK) located on chromosome 7p, and is activated via its natural ligand hepatocyte growth factor/scatter factor (HGF/SF) ligand. Sandra et al. previously observed MET gain in one ARMS cell line by aCGH [21]. In this study, we have also found MET amplification in RMS. Some results showed that MET possibly has a function not only in ARMS, which carries a dominant genetic lesion in an upstream transcription factor, but also in ERMS, in which the molecular mechanisms responsible for PAX3/PAX7 upregulation are more elusive [38], [39], [40]. The data of Riccardo indicated that MET may be necessary for RMS maintenance, and MET-directed therapies may be effective in the treatment of RMS [41]. Francesca et al. showed that MET is widely expressed in ARMS and ERMS at high levels in isolated marrow-infiltrating tumor cells. High levels of expression are associated with unfavorable clinical features, such as tumor marrow involvement [42]. The results of Hou possibly support the function of MET in the development and progression of RMS, and the inhibitor of MET can be an effective targeting therapy reagent for RMS, especially ARMS [43]. So, MET could play an important role in RMS and be expected to become a molecular therapeutic target.

HMGA2 is an important regulator of cell growth and differentiation. HMGA2 is expressed during embryogenesis, but is absent or presented at low levels in terminally differentiated tissues. Overexpression of HMGA2 is associated with aggressive tumor growth, early metastasis, and poor prognosis [44], [45], [46], [47]. Yang et al. showed that the expression of HMGA1 and HMGA2 in RMS with relapse or metastasis is higher than that in RMS without relapse or metastasis. Thus, the overexpression of HMGA1 and HMGA2 may be involved in the carcinogenesis and progression of RMS, and these two genes may also be prognostic indicators of the tumor and provide a new basis for targeted therapy [48]. Another report showed that HMGA2 is required for the proliferation and survival of ERMS cells both in vitro and in vivo [49]. In our study, the amplification of HMGA2 could have a co-effect function in RMS carcinogenesis.

In this study, we found that the gene deletion frequency of AMY2A was 20% in RMS, and we also found that AMY2A gene deletion could be involved in ERMS by functional annotation clustering. The AMY2A gene, which codes for salivary and pancreatic amylases, is located at 1p21.1 region. A homozygous deletion of the AMY2A gene is possibly involved in the pathogenesis of gastric carcinoma. AMY2A gene may have potential for tumor suppression in gastric carcinoma [50]. Mohammad showed that gene losses of AMY2A, TGFA, and REG1B in uterine leiomyosarcoma may be responsible for secondary changes that affect the progression and proliferation of the tumor [51]. At present, there are no studies that report AMY2A gene has role in RMS. It remains to be further studied whether AMY2A plays a role of tumor suppressor gene in RMS.

The participation of miRNAs in the pathogenesis of human cancer development has been suggested because dysfunction of specific miRNAs is associated with some cancers [52]. Multiple miRNAs are linked to oncogenes and tumor suppressor genes, including the Ras proto-oncogene, anti-apoptotic gene BCL2, potent p53 tumor suppressor gene, and MET oncogene. miRNAs act as tumor suppressors when they repress oncogenic genes, but act as oncogenes when they downregulate tumor suppressors [53]. In RMS, miR-1/206 suppresses MET expression and functions as a potent tumor suppressor in MET-overexpressing tumors [54]. TAM analysis showed upregulation of onco-miRNA (miR-24, miR-27a, and miR-146b). ALK4, MAPK14, and CDKN2A have been shown to be the target genes of multiple miRNAs, including miRNA-24 [55], [56], [57]. The CDKN2A gene is inactivated in numerous human tumors. Deletion of CDKN2A can lead to uncontrolled cell proliferation. Petra et al. reported that CDKN2A is an early event in urinary bladder transitional cell carcinoma [58]. These findings suggested the involvement of miRNAs in RMS.

In summary, this study has identified a number of differential changes in RMS-associated genetic alterations using aCGH and revealed several genes that may be candidate molecular targets for RMS. Taken together, the altered pathways may interact with one another in the induction of apoptosis, cell cycle, proto-oncogene, and amylase activity, all of which may ultimately contribute to the development and progression of RMS. Many of these implicated genes may be responsible for changes that affect tumor progression and proliferation. The findings presented here warrant further studies to investigate the pathophysiological functions of these candidate genes in RMS.
